# Delirium and Associated Length of Stay and Costs in Critically Ill Patients

**DOI:** 10.1155/2021/6612187

**Published:** 2021-04-24

**Authors:** Claudia Dziegielewski, Charlenn Skead, Toros Canturk, Colleen Webber, Shannon M. Fernando, Laura H. Thompson, Madison Foster, Vanja Ristovic, Peter G. Lawlor, Dipayan Chaudhuri, Chintan Dave, Brent Herritt, Shirley H. Bush, Salmaan Kanji, Peter Tanuseputro, Kednapa Thavorn, Erin Rosenberg, Kwadwo Kyeremanteng

**Affiliations:** ^1^Department of Medicine, University of Ottawa, Ottawa, ON, Canada; ^2^Ottawa Hospital Research Institute, University of Ottawa, Ottawa, ON, Canada; ^3^Bruyère Research Institute, Ottawa, ON, Canada; ^4^ICES uOttawa, Ottawa, ON, Canada; ^5^Division of Critical Care, Department of Medicine, University of Ottawa, Ottawa, ON, Canada; ^6^Department of Emergency Medicine, University of Ottawa, Ottawa, ON, Canada; ^7^Department of Anesthesia, University of Ottawa, Ottawa, ON, Canada; ^8^Division of Palliative Care, Department of Medicine, University of Ottawa, Ottawa, ON, Canada; ^9^Clinical Epidemiology Program, Ottawa Hospital Research Institute, Ottawa, ON, Canada; ^10^Department of Pharmacy, Ottawa, ON, Canada; ^11^School of Epidemiology and Public Health, Faculty of Medicine, University of Ottawa, Ottawa, ON, Canada; ^12^The Ottawa Methods Centre, Ottawa Hospital Research Institute, Ottawa, ON, Canada

## Abstract

**Purpose:**

Delirium frequently affects critically ill patients in the intensive care unit (ICU). The purpose of this study is to evaluate the impact of delirium on ICU and hospital length of stay (LOS) and perform a cost analysis.

**Materials and Methods:**

Prospective studies and randomized controlled trials of patients in the ICU with delirium published between January 1, 2015, and December 31, 2020, were evaluated. Outcome variables including ICU and hospital LOS were obtained, and ICU and hospital costs were derived from the respective LOS.

**Results:**

Forty-one studies met inclusion criteria. The mean difference of ICU LOS between patients with and without delirium was significant at 4.77 days (*p* < 0.001); for hospital LOS, this was significant at 6.67 days (*p* < 0.001). Cost data were extractable for 27 studies in which both ICU and hospital LOS were available. The mean difference of ICU costs between patients with and without delirium was significant at $3,921 (*p* < 0.001); for hospital costs, the mean difference was $5,936 (*p* < 0.001).

**Conclusion:**

ICU and hospital LOS and associated costs were significantly higher for patients with delirium, compared to those without delirium. Further research is necessary to elucidate other determinants of increased costs and cost-reducing strategies for critically ill patients with delirium. This can provide insight into the required resources for the prevention of delirium, which may contribute to decreasing healthcare expenditure while optimizing the quality of care.

## 1. Introduction

Delirium is defined as an acute, fluctuating disturbance in attention and awareness, with additional alterations in cognition, not explained by a preexisting neurocognitive disorder or generalized medical condition [1]. Delirium often occurs in the context of multiorgan failure and critical illness and therefore is common within the intensive care unit (ICU). Up to 40% of patients in the ICU experience delirium, of which 60–90% are mechanically ventilated [2–6]. Patients that experience delirium within the ICU have worse outcomes, including higher mortality, increased rates of mechanical ventilation, and longer length of stay (LOS) [4, 6–9].

Patient care for delirium in the ICU often involves frequent monitoring, extended hospitalization, and increased interventions, including diagnostic testing, pharmacological agents, restraints, and prolonged mechanical ventilation [10–17]. This likely translates into increased costs, which is supported by previous prospective studies demonstrating delirium is associated with up to 40% higher ICU and hospital costs, compared to patients without delirium [11, 12]. Therefore, prevention or early identification of delirium in the ICU may represent an area of optimizing healthcare spending and reducing costs. While previous review articles have analyzed the effect of delirium on clinically relevant ICU outcomes including LOS and mortality, no review articles to our knowledge have reviewed the influence of delirium on ICU costs [9, 18]. The purpose of this study is to evaluate the influence of delirium on ICU and hospital LOS and associated costs, in a narrative review and cost analysis.

## 2. Materials and Methods

### 2.1. Search Strategy and Selection Criteria

A narrative review and systematic literature search was conducted. We evaluated prospective observational studies and randomized controlled trials published between January 1, 2015, and December 31, 2020, in addition to studies published from 1966 to 2015 included in a previous review [9]. Databases including PubMed, EMBASE, CINAHL, The Cochrane Library, and PsycInfo were searched.

Studies with the following criteria were included: (1) observational prospective cohort studies or randomized controlled trials; (2) study population of adults (age ≥ 18 years) admitted to an ICU; (3) patients were evaluated for delirium using a validated screening or diagnostic instrument such as the Confusion Assessment Method (CAM), Confusion Assessment Method for the Intensive Care Unit (CAM-ICU), Intensive Care Delirium Screening Checklist (ICDSC), Diagnostic and Statistical Manual of Mental Disorders 4th and 5th edition (DSM-IV and DSM-V), Delirium Observation Screening Scale (DOS), or the Neelon and Champagne (NEECHAM) Confusion Scale; (4) outcomes measured included ICU LOS; and (5) articles were available in full text in English. Studies were excluded if (1) they had no comparison group of patients without delirium; (2) they were retrospective cohort or case series; (3) the largest subgroup of the patients had a primary central nervous system disorder (including stroke, traumatic brain injury, central nervous system infection, brain tumour, or recent intracranial surgery), were undergoing cardiac surgery or organ/tissue transplantation, were experiencing alcohol or substance withdrawal, or were diagnosed with COVID-19, since these are neurological processes considered to be distinct from delirium; or (6) the primary study endpoint was the comparative efficacy or safety of different sedative drugs.

### 2.2. Data Extraction

Two investigators (CD and CS) independently screened titles, abstracts, and full-text articles based on the above inclusion and exclusion criteria and extracted data from the relevant included studies. Discrepancies were handled through team discussion. Information was extracted using a standardized form, which included eligibility criteria, diagnostic tool used for identification of delirium, patient characteristics, illness severity, organ dysfunction scores, and outcomes (ICU and hospital LOS).

### 2.3. Statistical Analysis

The primary analysis compared the mean differences in hospital and ICU LOS and costs between patients with and without delirium. First, mean hospital and ICU LOS were obtained based on the summary statistics reported in the studies. For studies that reported median and interquartile ranges for the LOS, means were calculated using the method proposed by Wan et al. [19]. These were calculated for patients with and without delirium.

Hospital and ICU costs were derived by multiplying the mean LOS for delirious and nondelirious patients (across all included studies) by their respective costs per day, using the methodology by Kahn et al. and applied in other studies [20–22]. For estimated ICU costs, daily direct variable costs were as follows: day 1 $3,678, day 2 $1057, day 3 $839, day 4 $834, and day 5 $690 onward. Estimated hospital costs were calculated by using $249/day in addition to the total ICU cost [20]. Mean LOS was rounded up to the nearest day for these calculations. Direct variable costs, which exclude equipment, salaried labor, and other fixed costs, were used because they best reflect direct and immediate economic impact associated with reducing LOS [23, 24]. Costs were reported in USD with standard error and inflated to December 2020 prices according to the Consumer Price Index [25].

Means and mean differences in LOS and costs were calculated using the DerSimonian–Laird random-effects model with OpenMeta [Analyst] (version Yosemite Build, Centre for Evidence-Based Medicine, Brown School of Public Health, Providence, RI). *p* values <0.05 were considered statistically significant.

## 3. Results

The search strategy yielded 41 unique studies that met inclusion criteria [2, 13, 26–64]. A total of 117,255 patients were included in these studies, which represented 40 unique patient samples; on one occasion, a single patient population was reported in two separate articles [26, 27]. These data are represented in Table 1. Only one randomized controlled trial was available [29]. Delirium was diagnosed in 15,446 of 117,255 patients (13.2%). The most common tool for screening and diagnosis of delirium was CAM-ICU, which was used in 28 (68%) studies [2, 13, 29–43, 45–48, 56–61, 63, 64]. Other screening tools included the ICDSC (23%), DSM (5%), CAM (3%), and DOS (3%) [26–28, 44, 49–55, 62].

All studies reported ICU LOS. There were two studies for which mean LOS was combined from two patient groups: Ouimet et al. and Yamada et al., “No delirium” and “Subsyndromal delirium” groups were combined [26, 63]. The mean ICU LOS for patients with delirium was 9.40  ±  0.47 days, compared to a mean LOS of 3.39  ±  0.07 days for patients without delirium. The mean difference of the ICU LOS between patients with and without delirium was significant at 4.77 days (95% CI 3.94 to 5.60, *p* < 0.001). Of these studies, the hospital LOS was available for 27 studies. For patients with delirium, the mean hospital LOS was 22.3  ±  2.78 days, as compared to 16.0  ±  4.00 days for patients without delirium. The mean difference of hospital LOS between patients with and without delirium was significant at 6.67 days (95% CI 5.51 to 7.82, *p* < 0.001). These data are displayed in Table 2.

We calculated costs data for the 27 studies in which both ICU and hospital LOS were available, given that hospital costs were obtained directly from ICU costs. The mean ICU cost for patients with delirium was $12,935 ± $556, compared to a mean cost of $9,013  ±  $61 for patients without delirium. The mean difference of the ICU costs between patients with and without delirium was significant at $3,921 (95% CI $2,973 to $4,869, *p* < 0.001). For patients with delirium, the mean hospital cost was $20,236 ± $1,361, compared to a mean cost of $14,300 ± $1,267 for patients without delirium. The mean difference of the hospital costs between patients with and without delirium was significant at $5,936 (95% CI $4,663 to $7,209, *p* < 0.001).

## 4. Discussion

Delirium occurs frequently within the ICU and impacts the outcomes of critically ill patients, contributing to increased length of stay and mortality [4, 6–9]. We found that delirium in critically ill patients is associated with significantly higher ICU and hospital LOS, which has been supported by previous studies [4, 6–9, 29]. Patients with delirium often require prolonged mechanical ventilation and take longer to reach a cognitive and physical state that enables discharge from acute care [2–6, 65]. Taken together, this results in increased LOS and may explain the significantly higher LOS in the ICU and hospital found in our study for patients with delirium. Our cost analysis demonstrated that delirium is also correlated with significantly increased costs of approximately $5000 per admission, both within the ICU and hospital, which is a more novel addition to the literature. While the previous single-center, prospective studies have demonstrated that delirium is associated with increased ICU costs, this study reveals this on a larger scale and integrates hospital costs as well [11, 12]. These costs are likely to be cumulatively significant given the pervasiveness of delirium in the ICU [2–6].

In our study, increased costs are predominantly driven by prolonged LOS in the ICU, as hospital costs were derived from ICU LOS [66, 67]. In addition, patients with delirium often require increased interventions such as numerous investigations, increased nursing care, pharmacological and physical restraints, and treatments aimed at managing the underlying cause of delirium, which are all costly [2–9, 68]. We found that delirium prolonged LOS for patients by nearly one week, both within the hospital and ICU. Prior studies have demonstrated that there are especially high costs in the first week of developing delirium in the ICU, likely reflecting the increased need for procedural care and invasive mechanical ventilation in this timeframe [3, 4, 12].

The high cost of delirium should prompt evaluation into its prevention and early identification, as an opportunity to reduce healthcare expenditure. Recent studies have outlined recommendations for the prevention and management of delirium [69–71]. Measures that include optimization of sleep, mobility, and extended family visitation may reduce the risk of developing delirium while accruing minimal cost [70, 72, 73]. Screening tools and prediction models may identify delirium promptly and enable the implementation of early intervention and management [74, 75]. For example, there is evidence that adequate pain control and avoidance of certain triggers such as benzodiazepines promote the resolution of delirium [69]. This may shorten the duration of delirium and thereby reduce LOS and associated costs. There is no strong evidence supporting the use of pharmacologic agents to treat delirium in critically ill patients, and this may actually prolong delirium and increase costs [70]. Although dexmedetomidine was previously thought to reduce the duration of mechanical ventilation in patients with delirium, which could have reduced costs, recent literature suggests there is no difference in ventilator-free days or length of time without delirium, when compared to propofol [70, 76]. Given the high prevalence of delirium in critically ill patients, the above strategies may contribute to significantly reduced costs [2–6]. Furthermore, by enhancing the resolution of delirium, these methods can also reduce the risk of long-term cognitive impairment and mitigate the emotional burden of family members [77, 78].

There are several limitations to this study. Firstly, ICU and hospital costs were represented in USD and estimated and derived exclusively from LOS. Costs may vary by patient demographics, country, hospital protocols, and severity of illness, although there is evidence that delirium increases LOS even when adjusted for severity of illness [4, 9, 29]. Furthermore, increased short-term interventions may drive up costs independently of the LOS, which may underestimate total hospital costs. However, LOS has been previously found to be the greatest predictor of ICU costs, suggesting using this method is valid [79]. Finally, our inclusion criteria necessitated the use of delirium screening tools for diagnosis of delirium, which may have excluded some studies of patients with delirium.

## 5. Conclusions

Delirium in critically ill patients results in increased ICU and hospital LOS and costs. In this study, increased costs are largely driven by ICU LOS. Further research is required to determine other factors influencing ICU and hospital costs in patients with delirium, including increased investigations, monitoring, and treatments utilized. Taken together, these findings should prompt investment in the resources necessary for the prevention, early identification, and mitigation of delirium, which may contribute to a substantial reduction of healthcare expenditure.

## Figures and Tables

**Table 1:** Demographics and delirium screening tools of the included studies.

Study	Type of study	No. of enrolled patients	No. of patients with delirium (%)	Delirium screening tool	Physiologic scoring system	ICU LOS (days)	Hospital LOS (days)
Aldemir 2001	Prospective cohort	818	90 (11.0)	DSM-III	NR	10.7	15.6
Almeida 2014	Prospective cohort	170	161 (91.0)	CAM-ICU	SAPS II, SOFA	14.3	26.0
Angles 2008	Prospective cohort	69	41 (59.4)	CAM-ICU	NR	7.8	15.2
Balas 2009	Prospective cohort	114	34 (29.8)	CAM-ICU	APACHE II	8.7	17.4
Burry 2017	Prospective cohort	520	260 (50.0)	ICDSC	APACHE II	6.7	NR
Dittrich 2017	Prospective cohort	240	145 (60.4)	CAM-ICU	SAPS III	12.7	39.3
Falsini 2017	Prospective cohort	726	111 (15.3)	CAM-ICU	NR	2.8	7.3
Green 2019	Prospective cohort	455	160 (35.2)	CAM-ICU	APACHE II	5.5	11.7
Kenes 2017	Prospective cohort	70	53 (75.7)	ICDSC	APACHE II	9.0	13.0
Kim 2020	Prospective cohort	175	107 (61.1)	CAM-ICU	NR	21.7	40.9
Klouwenberg 2015	Prospective cohort	1112	535 (48.1)	CAM-ICU	APACHE IV, SOFA	10.7	NR
Lat 2009	Prospective cohort	134	84 (62.7)	CAM-ICU	APACHE II	11.0	18.8
Li 2017	Prospective cohort	336	102 (30.4)	CAM-ICU	APACHE II	11.2	NR
Lin 2008	Prospective cohort	151	31 (20.5)	CAM-ICU	APACHE III	16.5	34.3
Marquis 2007	Prospective cohort	537	189 (35.2)	ICDSC	APACHE II	10.8	36.4
Mehta 2015	RCT	420	226 (53.8)	ICDSC	APACHE II	14.3	29.7
Micek 2005	Prospective cohort	93	44 (47.3)	CAM-ICU	APACHE II	11.5	18.4
Ouimet 2007	Prospective cohort	764	243 (31.8)	ICDSC	APACHE II	11.5	18.2
Pauley 2015	Prospective cohort	590	120 (20.3)	CAM-ICU	APACHE II, SAPS II	5.7	NR
Pipanmekaporn 2015	Prospective cohort	4450	162 (3.64)	ICDSC	APACHE II, SOFA	10.7	23.3
Plaschke 2007	Prospective cohort	37	17 (46.0)	CAM-ICU	APACHE II	6.9	22.3
Roberts 2005	Prospective cohort	185	84 (45.4)	ICDSC	APACHE II	10.0	23.3
Salluh 2010	Prospective prevalence	232	75 (32.3)	CAM-ICU	SAPS III	24.3	NR
Sánchez-Hurtado 2018	Prospective cohort	109	25 (22.9)	CAM-ICU	NR	7.5	NR
Schubert 2018	Prospective cohort	10,906	3069 (28.1)	ICDSC	NR	4.4	40.3
Serafim 2012	Prospective cohort	467	43 (9.20)	CAM	APACHE II	7.3	25.7
Sharma 2012	Prospective cohort	140	75 (54.0)	DSM-IV	APACHE II	8.5	NR
Shehabi 2010	Prospective cohort	354	228 (64.4)	CAM-ICU	NR	15.3	NR
Singh 2018	Prospective cohort	67,333	1985 (2.95)	CAM-ICU	APACHE III, SOFA	1.4	8.1
Spronk 2009	Prospective cohort	46	23 (50.0)	CAM-ICU	APACHE II	13.7	30.3
Thomason 2005	Prospective cohort	261	125 (47.9)	CAM-ICU	APACHE II	4.0	5.0
Tilouche 2018	Prospective cohort	206	39 (18.9)	CAM-ICU	SAPS II	21.5	NR
Tsuruta 2010	Prospective cohort	103	21 (20.4)	CAM-ICU	APACHE II	13.3	NR
Van den Boogaard 2010	Prospective cohort	1740	332 (19.1)	CAM-ICU	APACHE II	4.3	18.7
Van den Boogaard 2012	Prospective cohort	1613	411 (26.0)	CAM-ICU	APACHE II	7.0	16.7
Van Rompaey 2008	Prospective cohort	172	34 (19.8)	CAM-ICU	NR	17.5	NR
Visser 2015	Prospective cohort	463	22 (4.75)	DOS	NR	3.0	14.0
Wolters 2014	Prospective cohort	1101	412 (37.0)	CAM-ICU	APACHE IV, SOFA	9.3	NR
Wood 2017	Prospective cohort	88	19 (21.6)	CAM-ICU	APACHE	11.7	NR
Yamada 2018	Prospective cohort	380	60 (15.8)	CAM-ICU	APACHE II	4.0	NR
Yamaguchi 2014	Prospective cohort	126	35 (27.8)	ICDSC	NR	7.1	36.3

A description of all included studies, according to primary author and year published. ICU and hospital LOS are reported for patients with delirium per study. RCT = randomized controlled trial; DSM = Diagnostic and Statistical Manual of Mental Disorders; ICDSC = Intensive Care Delirium Screening Checklist; CAM = Confusion Assessment Method; CAM-ICU = Confusion Assessment Method for the Intensive Care Unit; IQCODE = Informant Questionnaire on Cognitive Decline in the Elderly; DOS = Delirium Observation Screening Scale; APACHE = Acute Physiology and Chronic Health Evaluation Score; SOFA = Sequential Organ Failure Assessment Score; SAPS = Simplified Acute Physiology Score; NR = no response.

**Table 2:** Summary of ICU and hospital length of stay and associated costs for patients with and without delirium.

	Delirium	No delirium	Mean difference	Lower border (95% CI)	Upper border (95% CI)
ICU LOS	9.40 days	3.39 days	4.77 days	3.94 days	5.60 days
Hospital LOS	22.3 days	16.0 days	6.67 days	5.51 days	7.82 days
ICU costs	$12,935	$9,013	$3,921	$2,973	$4,869
Hospital costs	$20,236	$14,300	$5,936	$4,663	$7,209

Costs are represented in USD. Mean LOS and costs are displayed for patients with delirium and without delirium. Mean differences with lower and upper borders of the CI are displayed. CI = confidence interval.

## Data Availability

All data generated or analyzed during this study are included in this published article.

## References

[1] American Psychiatric Association (2013). *Diagnostic and Statistical Manual of Mental Disorders*.

[2] Tilouche N., Hassen M. F., Ali H. B. S., Jaoued O., Gharbi R., El Atrous S. S. (2018). Delirium in the intensive care unit: incidence, risk factors, and impact on outcome. *Indian Journal of Critical Care Medicine*.

[3] Ely E. W., Inouye S. K., Bernard G. R. (2001). Delirium in mechanically ventilated patients. *JAMA*.

[4] Ely E. W., Shintani A., Truman B. (2004). Delirium as a predictor of mortality in mechanically ventilated patients in the intensive care unit. *JAMA*.

[5] Pandharipande P., Cotton B. A., Shintani A. (2008). Prevalence and risk factors for development of delirium in surgical and trauma intensive care unit patients. *Journal of Trauma: Injury, Infection & Critical Care*.

[6] McPherson J. A., Wagner C. E., Boehm L. M. (2013). Delirium in the cardiovascular ICU. *Critical Care Medicine*.

[7] Lin S.-M., Liu C.-Y., Wang C.-H. (2004). The impact of delirium on the survival of mechanically ventilated patients. *Critical Care Medicine*.

[8] Pompei P., Foreman M., Rudberg M. A., Inouye S. K., Braund V., Cassel C. K. (1994). Delirium in hospitalized older persons: outcomes and predictors. *Journal of the American Geriatrics Society*.

[9] Salluh J. I. F., Wang H., Schneider E. B. (2015). Outcome of delirium in critically ill patients: systematic review and meta-analysis. *BMJ*.

[10] Pandharipande P. P., Girard T. D., Jackson J. C. (2013). Long-term cognitive impairment after critical illness. *New England Journal of Medicine*.

[11] Milbrandt E. B., Deppen S., Harrison P. L. (2004). Costs associated with delirium in mechanically ventilated patients. *Critical Care Medicine*.

[12] Vasilevskis E. E., Chandrasekhar R., Holtze C. H. (2018). The cost of ICU delirium and coma in the intensive care unit patient. *Medical Care*.

[13] Lin S.-M., Huang C.-D., Liu C.-Y. (2008). Risk factors for the development of early-onset delirium and the subsequent clinical outcome in mechanically ventilated patients. *Journal of Critical Care*.

[14] Ely E., Gautam S., Margolin R. (2001). The impact of delirium in the intensive care unit on hospital length of stay. *Intensive Care Medicine*.

[15] Girard T. D., Pandharipande P. P., Carson S. S. (2010). Feasibility, efficacy, and safety of antipsychotics for intensive care unit delirium: the MIND randomized, placebo-controlled trial. *Critical Care Medicine*.

[16] Tian J., Chen X., Liu D. (2018). Prediction of length of hospital stay and mortality in patients with delirium: a prospective cohort analysis of 200 ICU patients. *Journal of Biological Regulators and Homeostatic Agents*.

[17] Dasta J. F., McLaughlin T. P., Mody S. H., Piech C. T. (2005). Daily cost of an intensive care unit day: the contribution of mechanical ventilation. *Critical Care Medicine*.

[18] Krewulak K. D., Stelfox H. T., Ely E. W., Fiest K. M. (2020). Risk factors and outcomes among delirium subtypes in adult ICUs: a systematic review. *Journal of Critical Care*.

[19] Wan X., Wang W., Liu J., Tong T. (2014). Estimating the sample mean and standard deviation from the sample size, median, range and/or interquartile range. *BMC Medical Research Methodology*.

[20] Kahn J. G., Kronick R., Kreger M., Gans D. N. (2005). The cost of health insurance administration in California: estimates for insurers, physicians, and hospitals. *Health Affairs*.

[21] Kyeremanteng K., Gagnon L.-P., Thavorn K., Heyland D., D’Egidio G. (2016). The impact of palliative care consultation in the ICU on length of stay. *Journal of Intensive Care Medicine*.

[22] Chaudhuri D., Herritt B., Heyland D. (2017). Early renal replacement therapy versus standard care in the ICU: a systematic review, meta-analysis, and cost analysis. *Journal of Intensive Care Medicine*.

[23] Wunsch H., Gershengorn H., Scales D. C. (2012). Economics of ICU organization and management. *Critical Care Clinics*.

[24] Khandelwal N., Brumback L. C., Halpern S. D., Coe N. B., Brumback B., Curtis J. R. (2017). Evaluating the economic impact of palliative and end-of-life care interventions on intensive care unit utilization and costs from the hospital and healthcare system perspective. *Journal of Palliative Medicine*.

[25] (June 2020). Consumer price index: U.S. Bureau of Labor Statistics. https://www.bls.gov/cpi/tables.htm.

[26] Ouimet S., Kavanagh B. P., Gottfried S. B., Skrobik Y. (2007). Incidence, risk factors and consequences of ICU delirium. *Intensive Care Medicine*.

[27] Marquis F., Ouimet S., Riker R., Cossette M., Skrobik Y. (2007). Individual delirium symptoms: do they matter?. *Critical Care Medicine*.

[28] Serafim R. B., Dutra M. F., Saddy F. (2012). Delirium in postoperative nonventilated intensive care patients: risk factors and outcomes. *Ann Intensive Care*.

[29] Mehta S., Cook D., Devlin J. W. (2015). Prevalence, risk factors, and outcomes of delirium in mechanically ventilated adults. *Critical Care Medicine*.

[30] Klouwenberg P. M., Zaal I. J., Spitoni C. (2014). The attributable mortality of delirium in critically ill patients: prospective cohort study. *BMJ*.

[31] Pauley E., Lishmanov A., Schumann S., Gala G. J., van Diepen S., Katz J. N. (2015). Delirium is a robust predictor of morbidity and mortality among critically ill patients treated in the cardiac intensive care unit. *American Heart Journal*.

[32] Angles E. M., Robinson T. N., Biffl W. L. (2008). Risk factors for delirium after major trauma. *The American Journal of Surgery*.

[33] Shehabi Y., Riker R. R., Bokesch P. M., Wisemandle W., Shintani A., Ely E. W. (2010). Delirium duration and mortality in lightly sedated, mechanically ventilated intensive care patients. *Critical Care Medicine*.

[34] Lat I., McMillian W., Taylor S. (2009). The impact of delirium on clinical outcomes in mechanically ventilated surgical and trauma patients. *Critical Care Medicine*.

[35] Van den Boogaard M., Peters S. A., van der Hoeven J. G. (2010). The impact of delirium on the prediction of in-hospital mortality in intensive care patients. *Critical Care*.

[36] Tsuruta R., Nakahara T., Miyauchi T. (2010). Prevalence and associated factors for delirium in critically ill patients at a Japanese intensive care unit. *General Hospital Psychiatry*.

[37] Van Rompaey B., Schuurmans M. J., Shortridge-Baggett L. M., Truijen S., Elseviers M., Bossaert L. (2008). A comparison of the CAM-ICU and the NEECHAM Confusion Scale in intensive care delirium assessment: an observational study in non-intubated patients. *Critical Care*.

[38] Almeida I. C., Soares M., Bozza F. A. (2014). The impact of acute brain dysfunction in the outcomes of mechanically ventilated cancer patients. *PLoS One*.

[39] Van den Boogaard M., Schoonhoven L., Evers A. W. M., van der Hoeven J. G., van Achterberg T., Pickkers P. (2012). Delirium in critically ill patients. *Critical Care Medicine*.

[40] Balas M. C., Happ M. B., Yang W., Chelluri L., Richmond T. (2009). Outcomes associated with delirium in older patients in surgical ICUs. *Chest*.

[41] Dittrich T., Tschudin-Sutter S., Widmer A. F., Rüegg S., Marsch S., Sutter R. (2016). Risk factors for new-onset delirium in patients with bloodstream infections: independent and quantitative effect of catheters and drainages—a four-year cohort study. *Ann Intensive Care*.

[42] Li G., Lei X., Ai C., Li T., Chen Z. (2017). Low plasma leptin level at admission predicts delirium in critically ill patients: a prospective cohort study. *Peptides*.

[43] Plaschke K., Von Haken R., Scholz M. (2008). Comparison of the confusion assessment method for the intensive care unit (CAM-ICU) with the Intensive Care Delirium Screening Checklist (ICDSC) for delirium in critical care patients gives high agreement rate(s). *Intensive Care Medicine*.

[44] Visser L., Prent A., van der Laan M. J. (2015). Predicting postoperative delirium after vascular surgical procedures. *Journal of Vascular Surgery*.

[45] Wood M. D., Maslove D. M., Muscedere J. G., Day A. G., Gordon Boyd J. (2017). Low brain tissue oxygenation contributes to the development of delirium in critically ill patients: a prospective observational study. *Journal of Critical Care*.

[46] Green C., Bonavia W., Toh C., Tiruvoipati R. (2019). Prediction of ICU delirium. *Critical Care Medicine*.

[47] Sánchez-Hurtado L. A., Hernández-Sánchez N., Moral-Armengol D. (2018). Incidence of delirium in critically ill cancer patients. *Pain Research and Management*.

[48] Singh T. D., O’Horo J. C., Gajic O. (2018). Risk factors and outcomes of critically ill patients with acute brain failure: a novel end point. *Journal of Critical Care*.

[49] Yamaguchi T., Tsukioka E., Kishi Y. (2014). Outcomes after delirium in a Japanese intensive care unit. *General Hospital Psychiatry*.

[50] Roberts B., Rickard C. M., Rajbhandari D. (2005). Multicentre study of delirium in ICU patients using a simple screening tool. *Australian Critical Care*.

[51] Sharma A., Malhotra S., Grover S., Jindal S. K. (2012). Incidence, prevalence, risk factor and outcome of delirium in intensive care unit: a study from India. *General Hospital Psychiatry*.

[52] Kenes M. T., Stollings J. L., Wang L., Girard T. D., Ely E. W., Pandharipande P. P. (2017). Persistence of delirium after cessation of sedatives and analgesics and impact on clinical outcomes in critically ill patients. *Pharmacotherapy: The Journal of Human Pharmacology and Drug Therapy*.

[53] Pipanmekaporn T., Chittawatanarat K., Chaiwat O. (2015). Incidence and risk factors of delirium in multi-center Thai surgical intensive care units: a prospective cohort study. *Journal of Intensive Care*.

[54] Schubert M., Schürch R., Boettger S. (2018). A hospital-wide evaluation of delirium prevalence and outcomes in acute care patients-a cohort study. *BMC Health Services Research*.

[55] Aldemir M., Özen S., Kara I. H., Sir A., Baç B. (2001). Predisposing factors for delirium in the surgical intensive care unit. *Critical Care*.

[56] Falsini G., Grotti S., Porto I. (2018). Long-term prognostic value of delirium in elderly patients with acute cardiac diseases admitted to two cardiac intensive care units: a prospective study (DELIRIUM CORDIS). *European Heart Journal: Acute Cardiovascular Care*.

[57] Salluh J. I., Soares M., Teles J. M. (2010). Delirium epidemiology in critical care (DECCA): an international study. *Critical Care*.

[58] Spronk P. E., Riekerk B., Hofhuis J., Rommes J. H. (2009). Occurrence of delirium is severely underestimated in the ICU during daily care. *Intensive Care Medicine*.

[59] Thomason J. W., Shintani A., Peterson J. F., Pun B. T., Jackson J. C., Ely E. W. (2005). Intensive care unit delirium is an independent predictor of longer hospital stay: a prospective analysis of 261 non-ventilated patients. *Critical Care*.

[60] Wolters A. E., van Dijk D., Pasma W. (2014). Long-term outcome of delirium during intensive care unit stay in survivors of critical illness: a prospective cohort study. *Critical Care*.

[61] Micek S. T., Anand N. J., Laible B. R., Shannon W. D., Kollef M. H. (2005). Delirium as detected by the CAM-ICU predicts restraint use among mechanically ventilated medical patients. *Critical Care Medicine*.

[62] Burry L. D., Williamson D. R., Mehta S. (2017). Delirium and exposure to psychoactive medications in critically ill adults: a multi-centre observational study. *Journal of Critical Care*.

[63] Yamada C., Iwawaki Y., Harada K., Fukui M., Morimoto M., Yamanaka R. (2018). Frequency and risk factors for subsyndromal delirium in an intensive care unit. *Intensive and Critical Care Nursing*.

[64] Kim Y., Jin Y., Jin T., Lee S.-M. (2020). Risk factors and outcomes of sepsis-associated delirium in intensive care unit patients: a secondary data analysis. *Intensive and Critical Care Nursing*.

[65] Grover S., Avasthi A. (2018). Clinical practice guidelines for management of delirium in elderly. *Indian Journal of Psychiatry*.

[66] Kyeremanteng K., Wan C., D’Egidio G., Neilipovitz D. (2016). Approach to economic analysis in critical care. *Journal of Critical Care*.

[67] McLaughlin A. M., Hardt J., Canavan J. B., Donnelly M. B. (2009). Determining the economic cost of ICU treatment: a prospective “micro-costing” study. *Intensive Care Medicine*.

[68] Hunter A., Johnson L., Coustasse A. (2014). Reduction of intensive care unit length of stay. *The Health Care Manager*.

[69] Devlin J. W., Skrobik Y., Gélinas C. (2018). Clinical practice guidelines for the prevention and management of pain, agitation/sedation, delirium, immobility, and sleep disruption in adult patients in the ICU. *Critical Care Medicine*.

[70] Park S. Y., Lee H. B. (2019). Prevention and management of delirium in critically ill adult patients in the intensive care unit: a review based on the 2018 PADIS guidelines. *Acute and Critical Care*.

[71] Trogrlić Z., van der Jagt M., Bakker J. (2015). A systematic review of implementation strategies for assessment, prevention, and management of ICU delirium and their effect on clinical outcomes. *Critical Care*.

[72] Rosa R. G., Tonietto T. F., da Silva D. B. (2017). Effectiveness and safety of an extended ICU visitation model for delirium prevention. *Critical Care Medicine*.

[73] Kamdar B. B., Yang J., King L. M. (2013). Developing, implementing, and evaluating a multifaceted quality improvement intervention to promote sleep in an ICU. *American Journal of Medical Quality*.

[74] Schweickert W. D., Pohlman M. C., Pohlman A. S. (2009). Early physical and occupational therapy in mechanically ventilated, critically ill patients: a randomised controlled trial. *The Lancet*.

[75] Wassenaar A., van den Boogaard M., van Achterberg T. (2015). Multinational development and validation of an early prediction model for delirium in ICU patients. *Intensive Care Medicine*.

[76] Van den Boogaard M., Schoonhoven L., Maseda E. (2014). Recalibration of the delirium prediction model for ICU patients (PRE-DELIRIC): a multinational observational study. *Intensive Care Medicine*.

[77] Hughes C. G., Mailloux P. T., Devlin J. W. (2021). Dexmedetomidine or propofol for sedation in mechanically ventilated adults with sepsis. *The New England Journal of Medicine*.

[78] Serrano P., Kheir Y. N. P., Wang S., Khan S., Scheunemann L., Khan B. (2019). Aging and postintensive care syndrome- family: a critical need for geriatric psychiatry. *The American Journal of Geriatric Psychiatry*.

[79] Jackson J. C., Mitchell N., Hopkins R. O. (2015). Cognitive functioning, mental health, and quality of life in ICU survivors: an overview. *Psychiatric Clinics of North America*.

[80] Shorr A. F. (2002). An update on cost-effectiveness analysis in critical care. *Current Opinion in Critical Care*.

